# Beta cells in type 1 diabetes: mass and function; sleeping or dead?

**DOI:** 10.1007/s00125-019-4822-4

**Published:** 2019-02-14

**Authors:** Richard A. Oram, Emily K. Sims, Carmella Evans-Molina

**Affiliations:** 1 RILD Level 3, Institute of Biomedical and Clinical Science, University of Exeter Medical School, Royal Devon and Exeter Hospital, Barrack Road, Exeter EX2 5DW, UK; 2 NIHR Exeter Clinical Research Facility, University of Exeter Medical School, Exeter, UK; 3 The Academic Renal Unit, Royal Devon and Exeter NHS Foundation Trust, Exeter, UK; 4 Department of Pediatrics, Indiana University School of Medicine, Indianapolis, IN, USA; 5 The Herman B Wells Center for Pediatric Research, Indiana University School of Medicine, Indianapolis, IN, USA; 6 Department of Medicine, Indiana University School of Medicine, 635 Barnhill Drive, MS 2031A, Indianapolis, IN 46202, USA; 7 Roudebush VA Medical Center, Indianapolis, IN, USA

**Keywords:** Beta cell function, Beta cell mass, C-peptide, Network for Pancreatic Organ Donors with Diabetes, Proinsulin, Review, Type 1 diabetes

## Abstract

Histological analysis of donor pancreases coupled with measurement of serum C-peptide in clinical cohorts has challenged the idea that all beta cells are eventually destroyed in type 1 diabetes. These findings have raised a number of questions regarding how the remaining beta cells have escaped immune destruction, whether pools of ‘sleeping’ or dysfunctional beta cells could be rejuvenated and whether there is potential for new growth of beta cells. In this Review, we describe histological and in vivo evidence of persistent beta cells in type 1 diabetes and discuss the limitations of current methods to distinguish underlying beta cell mass in comparison with beta cell function. We highlight that evidence for new beta cell growth in humans many years from diagnosis is limited, and that this growth may be very minimal if at all present. We review recent contributions to the debate around beta cell abnormalities contributing to the pathogenesis of type 1 diabetes. We also discuss evidence for restoration of beta cell function, as opposed to mass, in recent-onset type 1 diabetes, but highlight the absence of data supporting functional recovery in the setting of long-duration diabetes. Finally, future areas of research are suggested to help resolve the source and phenotype of residual beta cells that persist in some, but not all, people with type 1 diabetes.

## Introduction

**Fig. 1:**
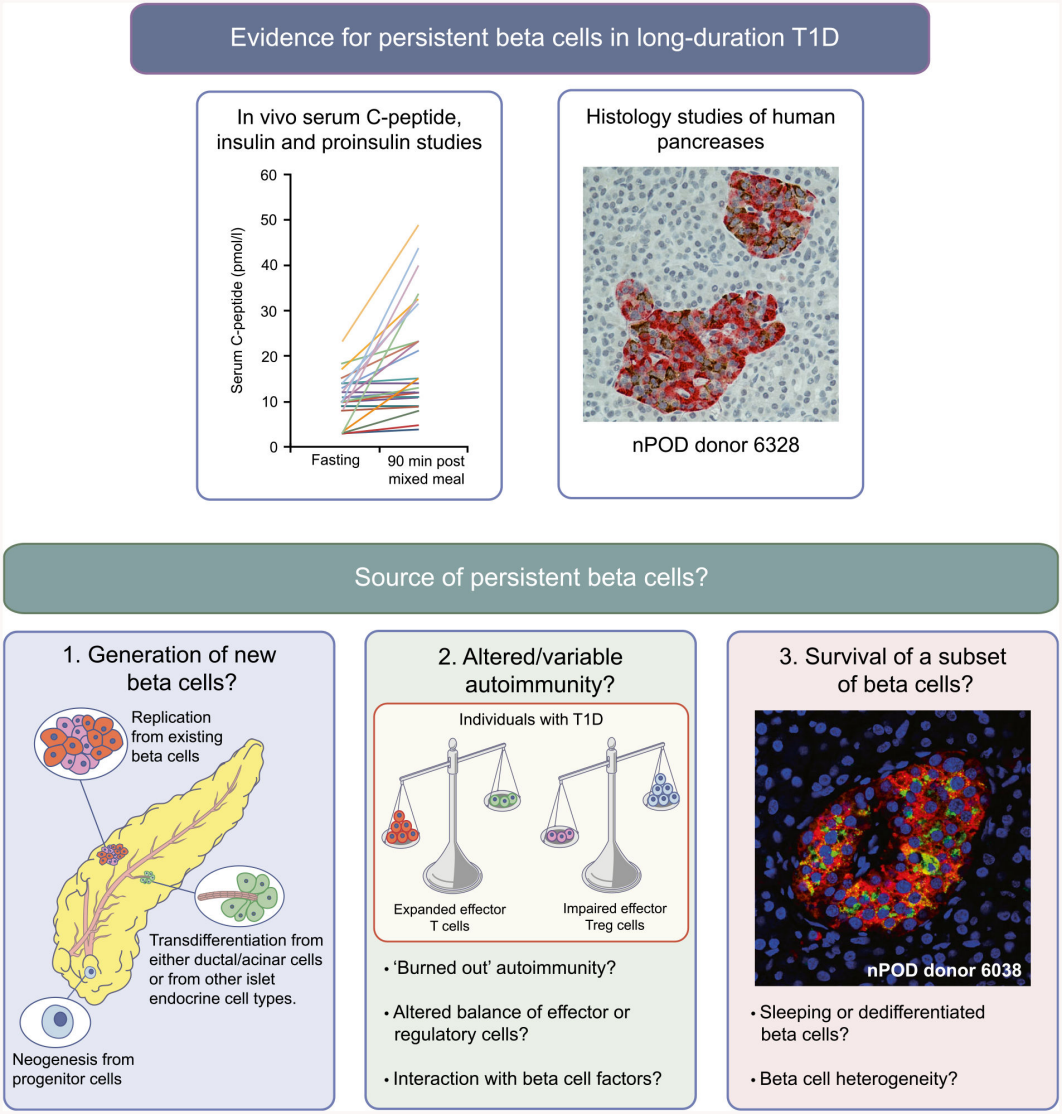
Persistent beta cells in type 1 diabetes. Histological and in vivo analyses (e.g. serum C-peptide, insulin and proinsulin studies) have provided evidence for the persistence of insulin-producing beta cells in long-duration type 1 diabetes. Potential explanations for this phenomenon include: (1) new beta cell growth via neogenesis, transdifferentiation and cell regeneration/turnover; (2) variation in intensity of autoimmune response, e.g. regulation of immune responses or a diminishing immune response may reduce beta cell destruction; and (3) that heterogeneity of beta cells may lead to beta cell protection. The histological image for nPOD donor 6328 was provided by S. J. Richardson (personal communication; University of Exeter Medical School, Exeter, UK) and shows an insulin-containing pancreatic islet from a 39-year-old organ donor with type 1 diabetes for 20 years (diagnosed aged 19). Insulin is stained in brown and glucagon in red. The histological image for nPOD donor 6038 was provided by T. L. Mastracci (personal communication; Indiana Biosciences Research Institute and the Indiana University School of Medicine, Indianapolis, IN, USA) and shows a pancreatic islet from an individual with type 1 diabetes for 20 years, in which beta cells have not been lost and continue to express both proinsulin and insulin (proinsulin, green; insulin, red; DAPI, blue [nuclei]). T1D, type 1 diabetes. Copyright details: the serum C-peptide graph is derived from [2], published under the terms of the Creative Commons Attribution 4.0 International License (http://creativecommons.Org/licenses/by/4.0/), which permits unrestricted use, distribution, and reproduction in any medium; the image for the generation of new beta cells is from [19], adapted by permission from Springer Nature © 2005; the image for altered/variable autoimmunity is from [20], adapted by permission from Springer Nature © 2014. This figure is available as a downloadable slide

Recent research has challenged the dogma that all beta cells are eventually destroyed in type 1 diabetes. This stems from the observation that some individuals with long-duration disease retain measurable levels of serum C-peptide [1–7] and exhibit the persistence of insulin-positive islets even decades after diagnosis [1,8–14]. These findings have shifted our understanding away from the model of complete and inevitable beta cell destruction that is described in many textbooks as part of the Eisenbarth model of type 1 diabetes [15–18]. They have also raised a number of fundamental questions regarding the trajectory of beta cell loss, the source of residual beta cells, whether a pool of ‘sleeping’ or dysfunctional beta cells could be enhanced or rejuvenated and whether there is potential for new growth of beta cells (Fig. 1). Here, we describe the human evidence behind some of these issues, highlight current areas of uncertainty and suggest future areas that should be addressed by the research community.

## Histological and in vivo analyses provide evidence for the persistence of insulin-producing beta cells in long-duration type 1 diabetes

It is not possible to routinely collect pancreatic samples from living individuals with type 1 diabetes. Thus, our histological knowledge of human type 1 diabetes comes from analyses of post-mortem pancreatic specimens, which are limited by specimen availability and the cross-sectional nature of autopsy studies. Notwithstanding these limitations, the presence of insulin containing islets (ICIs) in long-duration type 1 diabetes was described in the literature as early as 1959. This has been corroborated in a number of additional cohorts, including single-centre collections and more contemporary efforts from the JDRF Network of Pancreatic Organ Donors (nPOD) programme, which was initiated 10 years ago to collect high-quality specimens from individuals with autoantibody positivity, recent-onset disease, long-duration disease and nondiabetic control donors [21]. Table 1 summarises findings from a selection of these major collections [1, 8–13, 23].

A number of themes are evident from the published literature. First, beta cell mass is markedly heterogeneous in people with type 1 diabetes and even amongst those without diabetes [24–27]. Beta cell mass at type 1 diabetes onset varies and may not match severity of clinical presentation [10,25,28]. In longstanding type 1 diabetes, beta cells can be observed in a significant proportion of donors, but overall beta cell mass is markedly reduced [1, 9, 11–13, 22, 23]. For example, a recent analysis of 47 nPOD donors with a disease duration ranging from 0 to 41 years found that 67% of donors had demonstrable ICIs. However, even in those with remaining ICIs, total beta cell mass was reduced by an estimated 88–95% [11]. Consistent with serum C-peptide analyses, there is data to suggest that ICIs are more likely to be observed in donors with an older age of diagnosis [29]. Other analyses describe a decline in beta cell area and mass with increasing disease duration [11]. Insulitis is common in individuals with disease duration <1 year, but immune infiltrates are not present in all islets within an affected individual [13]. In long-duration type 1 diabetes, insulitis may still be observed but is much rarer [13].

The progressive decline in beta cell function after type 1 diabetes diagnosis is described in numerous longitudinal studies of serum C-peptide post-diagnosis. Initial decline of beta cell function seems to follow a loglinear trajectory [30–34], with age of diagnosis significantly affecting C-peptide level at the time of diagnosis but perhaps having less impact on gradient of decline [31, 33]. However, it is important to note that some studies suggest the rate of decline in beta cell function post-diagnosis is accelerated in younger individuals [30, 34]. Emerging data indicate that decline in beta cell function may plateau after approximately 7 years [31], although there is still a relative paucity of longitudinal data on beta cell function in long-duration type 1 diabetes.

In parallel with histological observations, it is now clear that endogenous insulin, as measured by serum C-peptide, persists years after diagnosis in many people with type 1 diabetes. This observation is not new but has received renewed attention in recent years, in part due to improvements in sensitivity of C-peptide assays. As early as 1978, Madsbad et al made the observation that C-peptide was detectable in all individuals within 2 years of type 1 diabetes diagnosis and even in 15% of individuals with 15–35 years of disease duration [5]. Many of the >3000 individuals screened for inclusion in the Diabetes Control and Complications Trial (DCCT) had a mixed-meal stimulated C-peptide above 30 pmol/l, with higher C-peptide concentrations being more common in individuals diagnosed as adults (readers are referred to scatterplots of C-peptide in all screened individuals in [35]). An analysis of Joslin Medalists in 2010, all with over 50 years of type 1 diabetes, highlighted persistent C-peptide (>30 pmol/l) in most people (67%) and identified some individuals (2.6%) with clinically significant levels of C-peptide (>200 pmol/l), suggesting significant beta cell reserve. This work additionally showed the presence of insulin-positive cells in nine donor Medalist pancreases [1]. The potential survivor bias and select nature of this cohort made it difficult to generalise these findings. However, several large and more heterogeneous long-duration cohorts have since been analysed, using C-peptide assays with improved sensitivity and specificity [2–4, 6, 7, 36]. These analyses have shown that between 11% and 80% of people with long-duration type 1 diabetes have detectable C-peptide, most commonly at very low concentrations. Variation in these studies may be explained by cohort selection criteria, differing assay sensitivities, cohort ages and durations, and sample storage conditions. Low-level C-peptide can be meal responsive, suggesting low C-peptide concentrations are not due to assay noise and that persistent C-peptide at least partially reflects functional beta cells [2, 7]. The dynamics of insulin secretion in relation to physiological stimuli, and to diabetes complications, is less-well studied in people with long-duration type 1 diabetes.

## Dissecting persistent beta cell mass vs persistent beta cell function in long-standing type 1 diabetes

Findings from analysis of beta cells by histology and serum C-peptide measurement raise important questions about the relationship between beta cell function and mass at different stages of type 1 diabetes. Measures of beta cell mass are currently limited to morphometric analysis of insulin-positive cells in tissue sections from autopsy specimens [26]. Whilst there is interest in the development of non-invasive imaging techniques for assessment of beta cell mass in living individuals, none of the currently available methods have adequate sensitivity to detect such small numbers of residual beta cells in people with long-duration type 1 diabetes [37]. Assessment of beta cell function in living individuals is performed within the context of a specific physiological state (e.g. fasting or post stimulation), measuring insulin release from beta cells functioning at that specific moment. Insulin secretion in response to physiological stimuli can be monitored over time using a variety of methods, such as intravenous glucose tolerance testing and oral glucose and mixed-meal tolerance tests [38, 39]. More specialised tests, like glucose-potentiated arginine testing and glucagon administration post-hyperglycaemic clamp testing, provide insight into the more abstract concept of functional beta cell mass by achieving maximal stimulation of beta cell insulin release [40, 41].

Against this background, several lines of evidence suggest a disconnect between beta cell mass and function in early-stage and established type 1 diabetes. A recent cross-sectional study compared findings from intravenous glucose tolerance tests (IVGTTs) and glucose-potentiated arginine testing in autoantibody-positive individuals from the TrialNet Pathway to Prevention (PTP) cohort [42]. This analysis revealed significant variability in the acute insulin response to arginine in high-risk multiple-autoantibody-positive relatives with low first phase insulin post IVGTT in the PTP cohort. These findings highlight a disconnect between measures of mass and function that is present well before the onset of stage 3 (symptomatic) type 1 diabetes. Analysis of functional beta cell mass in recent-onset type 1 diabetes, using hyperglycaemic clamps followed by glucagon stimulation, has suggested that C-peptide secretion is 25% of that observed in people without diabetes [43, 44]. At the tissue level, individuals with new-onset type 1 diabetes have been described at autopsy as having 30–60% of ‘normal beta cell mass’ [10, 25], but this figure is very hard to estimate given the up to fourfold variation in beta cell mass between healthy individuals [27]. It is notable that these somewhat modest reductions in beta cell mass have been associated with death from severe insulin deficiency [10, 25] and, in individuals with long-established diabetes, abundant residual beta cells have occasionally been described in the context of low serum C-peptide levels [45]. Only one study from the nPOD repository has been able to correlate serum C-peptide with findings on histology; amongst 47 type 1 diabetes donors, serum C-peptide levels were positively correlated with both beta cell area and mass. Interestingly, no correlation was observed in control donors [11]. However, an important caveat of this study is that collection of samples for C-peptide analysis was typically performed at the time of organ donation, when donors were critically unwell.

## What is the source of persistent beta cells in long-duration type 1 diabetes?

A key question is whether beta cells that exist in those with long-duration type 1 diabetes are newly formed and whether this is a result of proliferation, regeneration or transdifferentiation. Whilst each of these processes has been shown to occur in mouse models of type 1 diabetes, differences exist between rodent and human beta cells, moderating enthusiasm for the existence of these processes in human type 1 diabetes [46, 47]. During early human development, beta cells are thought to arise either via neogenesis from progenitor cells and ductal precursor cells or through replication of existing beta cells [48]. Proliferation is identifiable in early childhood pancreatic samples but samples from people older than 5 years suggest that there is very little proliferation in later child- and adulthood [11, 46, 48]. Reports based on analysis of a small number of pancreatic sections have suggested there is evidence for beta cell proliferation in long-duration type 1 diabetes [1, 49]. However, a recent large and detailed analysis from the nPOD repository failed to find evidence of either proliferation or neogenesis [11]. Similarly, there is no direct human evidence to support the idea that new beta cells transdifferentiate from alpha cells or pancreatic ductal glands in long-standing type 1 diabetes [11,50]. Even direct evidence of beta cell death, using histological studies, has been difficult to demonstrate consistently [11, 23]. Recently, circulating cell-free unmethylated preproinsulin DNA (a proposed biomarker of beta cell death [51–53]) was found to be elevated in a cross-section of 90 individuals with long-standing type 1 diabetes [54]. Although this marker may theoretically allow for analysis of beta cell death in living individuals, given the limitations of the sensitivity and specificity of this assay, combined with the likely tiny number of cells dying at any one time, interpretation of these data are challenging without longitudinal analyses in the same individuals.

An alternative explanation for persistent beta cells in long-duration type 1 diabetes is that residual beta cells may survive because they have acquired an intrinsic ability to evade immune destruction. This is supported by recent studies using the NOD mouse model, where a protected population of cells was found to have reduced insulin content, decreased expression of genes associated with beta cell identity and reduced glucose stimulated insulin secretion. In parallel, genes associated with immune modulation and ‘sternness’ were increased [55]. The model put forth suggested that this protection from autoimmunity may be owing to the acquisition of a ‘de-differentiated’ phenotype. However, a consensus definition of this precise phenotype has yet to be established and proven in human pancreases, although the studies suggesting a disconnect between persistent beta cell mass and function may be consistent with the idea of beta cell de-differentiation in long-duration type 1 diabetes. Intriguingly, recent data show that the majority of individuals with long-duration diabetes retain the ability to secrete proinsulin, even those lacking measurable serum C-peptide [56]. At the tissue level, there is markedly reduced insulin content and dysfunctional hormone processing coupled with proinsulin accumulation in both the pancreas and in the circulation [57, 58]. This suggests that there are populations of beta cells that can initiate hormone production but may be unable to release mature insulin and C-peptide. Whether these findings corroborate the idea of de-differentiation and the presence of sleeping beta cells in long-standing type 1 diabetes remains an open question.

The idea that different populations of beta cells exist in those with long-duration type 1 diabetes intersects closely with the idea that type 1 diabetes is a heterogenous disease. Single-cell genomic analyses have uncovered marked heterogeneity amongst isolated human beta cells from non-diabetic cadaveric donors and identified subsets of beta cells with unique transcriptomic, cell-surface, and functional characteristics [59–61]. Moreover, data support the idea of histopathological heterogeneity in samples from individuals with long-duration type 1 diabetes, where some [10, 45, 62], but not all [11], analyses have described patchy, regional persistence of beta cells. Heterogeneity in the distribution of insulitis has been similarly described [11, 25, 45, 62] and there is significant heterogeneity in the cellular composition of insulitic lesions that appears to stratify with age of onset [29]. How this heterogeneity impacts antigenicity is still a matter of speculation. However, a causal role for beta cell abnormalities in type 1 diabetes pathogenesis, rather than just being a result of damage caused by immune destruction, has gained traction in recent years. Supporting evidence for this includes: increased beta cell expression of immune genes associated with type 1 diabetes, under inflammatory conditions [63]; beta cell HLA class I hyperexpression (a homing signal for T cells) in insulitis [64]; and the recent linking of endoplasmic reticulum stress with altered mRNA splicing and production of highly immunogenic neoantigens [65–67]. The studies yielding these findings have utilised isolated cadaveric islets and human beta cells treated with proinflammatory cytokines and other diabetogenic stressors. Thus, further experiments using human samples are needed to define truly whether remaining beta cells in longstanding type 1 diabetes are resistant to immune attack because of an intrinsic phenotypic switch or whether these beta cells persist primarily because the intensity of immune attack is reduced.

## Can beta cell function and/or mass be recovered in long-standing type 1 diabetes?

Is it possible that beta cell function and/or mass could be reparable in individuals with long-duration type 1 diabetes? Evidence for reversibility of beta cell dysfunction and insulin insufficiency comes from other clinical conditions, such as ketosis-prone diabetes, where affected individuals present with insulin deficiency severe enough to cause ketoacidosis, followed by recovery of beta cell function that often allows for discontinuation of exogenous insulin [68, 69]. In type 1 diabetes, there is often a ‘honeymoon period’ of partial clinical remission in the months after diagnosis, where newly diagnosed individuals often exhibit improved glycaemic control (HbA_lc_ <48 mmol/mol [<6.5%]) and reduced insulin dose requirements (<0.5 U/kg) [70–72]. This tends to occur in the first year after diagnosis and insulin initiation, lasting for months, and is assumed to be due to beta cell recovery from the acute effects of glucotoxicity and improved insulin sensitivity. Along these lines, improved metabolic control with intensive insulin therapy in the DCCT was associated with improved residual mixed-meal stimulated C-peptide values [73]. A recent study using beta cells that were isolated by biopsy from a small cohort of newly diagnosed adults clearly demonstrated that beta cell function could improve ex vivo when these cells are removed from an in vivo diabetogenic environment for a period of days [74]. The time scale of this improvement strongly supports the notion that some functional beta cell recovery is possible in people close to diagnosis.

Although the possibility of functional recovery of beta cells is tantalising, there is very limited evidence of functional recovery of beta cells outside of the honeymoon period. Compared with usual care, aggressive glycaemic control of new-onset type 1 diabetes patients with 71.3 h of inpatient hybrid closed-loop therapy, followed by sensor-augmented pump therapy, had no effect on honeymoon stimulated C-peptide values 12 months after diagnosis [75]. Pregnancy in women with type 1 diabetes is often cited as an example of when beta cell functional recovery or proliferation may occur. However, a small number of human studies in this area have provided conflicting results and it is yet to be resolved whether improvements in beta cell function or an increase in mass is possible during pregnancy [76–78]. Despite improvements in hyperglycaemia, islet transplant recipients with longstanding type 1 diabetes that achieve insulin independence often ultimately require resumption of insulin; although this picture is complicated by functional effects of transplant medications on beta cell health [79].

## Summary

This Review highlights evidence from functional human studies and pancreatic autopsy samples that persistent low-level beta cell function is relatively common in long-standing type 1 diabetes, particularly in people diagnosed as adults. A common feature of these studies is the small number of detectable beta cells and very low levels of endogenous insulin secretion. So, whilst the total number of beta cells in long-duration disease is vastly reduced, some persist and maintain the ability to produce and secrete insulin. These observations raise fundamental, yet currently unanswered, questions about the nature of these cells and whether certain subpopulations of beta cells are more resistant to immune destruction, whether there is a reduction in the intensity of autoimmunity over time, or whether new beta cells have the capacity to regrow. We have highlighted increasing awareness of a dynamic interaction between beta cells and the immune system that may contribute to type 1 diabetes pathogenesis and potential heterogeneity of beta cell loss in those with the disease. The identification of impaired insulin expression and proinsulin processing abnormalities also links to the question of whether some beta cells are sufficiently dysfunctional to be considered ‘sleeping’ and whether these cells could somehow be awoken. There is evidence for reversibility of beta cell dysfunction in specific settings, such as the honeymoon period shortly after diagnosis of type 1 diabetes and in relatively rare ketosis-prone diabetes. However, there is no evidence, as of yet, to suggest pools of sleeping beta cells are poised for rejuvenation in long-duration type 1 diabetes. Better longitudinal studies of long-standing type 1 diabetes, using robust, repeatable physiological assays paired with biomarkers of beta cell dysfunction and death, and continued interrogation of donor pancreatic samples may prove that we have not yet looked hard enough or applied the right tools for this search. This topic is important because most people with type 1 diabetes have some remaining beta cells and some rare individuals have a very large number of beta cells despite many years of type 1 diabetes. If we can understand the causes of this variation in beta cell destruction, we may be a step closer to preserving beta cell function in all individuals with type 1 diabetes.

## Figures and Tables

**Table 1:** Summary of type 1 diabetes histopathology collections

Study (year)	Location	Number of donors	Age at diagnosis, years (mean ± SD)	Diabetes duration (mean ± SD)	Major findings
Maclean and Ogilvie (1959) [9]	Not specified	*N* = 41 diabetic donors: RO: *n* = 26 LD: *n* = 15	RO: 14.3 ±7.5 LD: 13.6 ±5.3	RO: 261.5±400.3 days LD: 8.3 ± 4.5 years	All donors had some residual islet tissue. Islet size decreased in proportion to T1D duration.
Gepts and De Mey (1978) [22]	Belgium, France, USA	*N=* 58 T1D donors: RO: *n* = 18 LD: *n* = 40	RO: 11.5 ±9.0 LD: 13.9 ±7.3	RO: 55.7 ±89.8 days LD: 16.9 ±7.9 years	Beta cells present in 16/18 (89%) of people with RO and 10/40 (25%) of those with LD.
Löhr and Kloppel (1987) [12]	Not specified	*n* = 26 T1D donors, categorised by distribution of residual insulin+ cells^a^: 0: *n* = 13 1+: *n* = 5 2+: *n* = 8 *n* = 45 non-diabetic control donors	0: 23 ±11 1+: 22 ±24 2+: 21 ± 14	0: 30 ± 9 years 1+: 27 ± 19 years 2+: 20 ±12 years	50% of all cases had small clusters of islets, containing only a few insulin+ cells. No significant relationship between age of T1D onset and presence of residual beta cells. Weak inverse relationship between diabetes duration and beta cell persistence; most robust with diabetes duration <11 years. Reduced pancreatic volume in T1D vs control.
Meier et al (2005) [23]	USA	*n* = 42 T1D donors *n* = 14 non-diabetic control donors	ND	4–67 years^b^	Beta cells identified in 88% of donors with T1D. Ratio of beta cell area to exocrine area markedly reduced in T1D vs control (0.02 7 ± 0.01 *%* vs 1.140 ±0.90%; p< 0.0001). Number of residual beta cells not related to disease duration/age of death, but higher in those with lower mean blood glucose. Increased apoptosis of beta cells in T1D samples, but no difference in proliferation. In renal transplant recipients, who had received immunosuppressive therapy, frequency of beta cells was non-significantly reduced vs other donors with T1D.
Butler et al (2007) [10]	USA	*n =* 9 T1D donors, all RO *n* = 9 non-diabetic control donors	23.44 ±10.24	336.8±416.2 days	90% mean reduction in beta cell mass in T1D vs control (range: 70–99%). Lobular distribution of residual islets. No relationship between fractional beta cell area and T1D duration.
Keenan et al (2010) [1]	USA	*n* = 9 donors, from the Joslin Medalist Study	10.0 ±9.7	64.3 ±9.9 years	9/9 pancreases had some residual insulin+ cells. Most insulin+ cells were small clusters of cells or singlets. In *n* = 2 (age of onset 23 and 30), insulin+ cells were more prevalent and located clearly within islets. In one of these donors, insulin+ cells were distributed in a lobular pattern.
Lam et al (2017) [11]	USA	*n* = 47 T1D donors, from the nPOD repository: RO: *n =* 9 LD: *n* = 38 *n* = 59 non-diabetic control donors	RO: 14.1 ±7.0 LD: 24.8 ±11.2	RO: 1.6 ± 1.2 years LD: 13.5 ± 10.2 years	Beta cells present in 64% of all samples and 58% (22/38) of LD samples. Beta cell area and mass correlated with C-peptide in T1D but not in control group. Regional distribution present in some but not all T1D pancreases. Beta cell area and mass decreased with increasing disease duration. No evidence for beta proliferation, neogenesis or transdifferentiation in T1D. No increase in TUNEL+ beta cells.
Rodriguez-Calvo et al 2018 [13]^c^	Europe (EADB), USA (nPOD)	*n* = 128 T1D donors, from EADB *n* = 133 T1D donors, from nPOD	EADB: 11 (5–16)^d^ nPOD: 11.5 (6.2–18.4)	EADB: 0.14 years (0.04–3.75)^d^ nPOD: 12.0 years (5.5–23.0)	EADB cases > 1 year duration with residual ICIs, categorised by age of onset: <7 years: 10% (*n* = 20) ≥7–<13 years: 28.6% (*n* = 14) ≥13 years: 56.3% (*n* = 16). nPOD cases >1 year duration with residual ICIs, categorised by age of onset: <7 years: 15.6% (*n =* 32) 7–12 years: 25.8% (*n* = 31) ≥13 years: 46.9% (*n* = 49).

a Category 0, ICIs throughout the pancreas; category 1+, ICIs in one lobule; category 2+, ICIs in >1 lobule

b Range

c Summary statistics provided for cases from the Exeter Archival Diabetes Biobank and nPOD biorepositories; possible overlap with data shown in Lam et al (2007) [11], which reported on a subset of nPOD donors

d Median (interquartile range)

EADB, Exeter Archival Diabetes Biobank; Insulin+, insulin-positive; LD, long-duration type 1 diabetes (duration >3 years); ND, no data; RO, recent-onset type 1 diabetes (duration ≤ 3 years); T1D, type 1 diabetes

## Abbreviations

ICI: Insulin containing islet
nPOD: Network of Pancreatic Organ Donors
PTP: TrialNet Pathway to Prevention
